# Contaminated well water driving d‐lactic acidosis in child with short bowel syndrome

**DOI:** 10.1002/jpr3.12133

**Published:** 2024-09-25

**Authors:** Judy‐April Murayi, Colleen Flahive, Ethan Mezoff

**Affiliations:** ^1^ Division of Gastroenterology, Hepatology, and Nutrition, Department of Pediatrics Nationwide Children's Hospital Columbus Ohio USA

**Keywords:** enteral nutrition, intestinal failure, lactic acidosis, parenteral nutrition

## Abstract

d‐Lactic acidosis is a rare type of lactic acidosis that typically presents in patients with short bowel syndrome (SBS). Clinical features include a high anion‐gap metabolic acidosis and acute onset of neurological impairment. The underlying pathology is thought to be due to altered gut flora and carbohydrate malabsorption in patients with altered gut anatomy. The treatment centers on correcting acid–base derangements, dietary modifications to decrease carbohydrate intake and antibiotics. We present a case of recurrent d‐lactic acidosis in a patient with SBS. In this unique case, we highlight the importance of considering the home environment when developing a treatment plan.

## 1 CASE REPORT

A 6‐year‐old male with short bowel syndrome (SBS) related to volvulus presents to the emergency department (ED) with diarrhea and altered mental status. He has 22 cm of small bowel (5% of expected small bowel) with an ileocecal valve in continuity with a full colon.^1^ He receives a combination of parenteral nutrition (PN) via a tunneled cuffed central line, which provides 40% of his daily calorie needs, and standard pediatric formula with fiber administered orally and via gastrostomy tube. He also adheres to an oral SBS diet, which avoids simple sugars and sugar alcohols to prevent gas, bloating, and increased stool output.

Two months before his presentation, his parents noticed bloating, irritability, and fatigue. On evaluation, he had a mild metabolic acidosis and subsequently started 2‐week cycles of 5 mg/kg of metronidazole three times daily for presumed small bowel bacterial overgrowth (SBBO). Four days before his ED presentation, he was evaluated by his primary gastroenterologist for 3 days of loose non‐bloody stools without fever or sick contacts. Laboratory evaluation revealed a serum bicarbonate (HCO_3_) of 14 mmoL/L and chloride of 112 mmoL/L. Half sodium acetate (77 mEq/L) was therefore added to his dextrose‐containing fluids with a plan to repeat labs in a week.

He woke up confused and dizzy the morning of the presentation. He answered questions appropriately but with slow, slurred speech. His parents called emergency medical services. In the ED, his vital signs were normal. He had a normal neurological exam. Complete blood count was normal. Urinalysis was notable only for 30 mg/dL of protein. Basic metabolic panel was notable for a high anion‐gap (AG) metabolic acidosis (Table 1). Gastrointestinal (GI) stool polymerase chain reaction was positive for Norovirus. He received a normal saline bolus and reported feeling better. He was admitted to the Intestinal Rehabilitation Service for further management.

**Table 1:** Lab results.

Lab values	ED 10:56	Admission 14:50
**Sodium** Reference range: 135–145 mmol/L	138	139
**Potassium** Reference range: 3.6–4.9 mmol/L	4	3.8
**Chloride** Reference range: 98–110 mmol/L	115	111
**Carbon dioxide** Reference range: 21–30 mmol/L	10	13
**BUN** Reference range: 5–18 mg/dL	7	6
**Creatinine** Reference range: 0.3–0.6 mg/dL	0.34	0.34
**Glucose** Reference range: 60–115 mg/dL	101	96
**Calcium** Reference range: 8–10.5 mg/dL	9.4	9.5
**Anion gap** Reference range: 4–12 mmol/L	13	13

Abbreviations: BUN, blood urea nitrogen; dL, deciliter; ED, emergency department; L, liter; mg, milligram; mmol, millimole.

On admission, his home fluids were switched to full sodium acetate (154 mEq/L). Later that day, a nurse witnessed an abrupt change in his behavior characterized by slurred but coherent speech, dizziness, and ataxia. The medical team called for immediate pediatric intensive care unit evaluation. He underwent an extensive workup notable for a high AG metabolic acidosis (Table 1) with normal lactate and ammonia levels and normal brain magnetic resonance imaging. His acidosis resolved on full sodium acetate intravenous fluids. After 2 days, his d‐lactate returned elevated at 1.96 mmol/L (reference range: 0.0–0.25 mmol/L) confirming the diagnosis of d‐lactic acidosis.

Treatment of his d‐lactic acidosis included maintaining normal electrolytes through 25 mEq enteral sodium bicarbonate supplementation twice daily, adjusting electrolyte doses in PN, transitioning from metronidazole to 200 mg rifaximin three times daily for 3 weeks at a time for SBBO treatment, dietary carbohydrate restriction, elimination of fermented foods and switching to Pediasure 1.5 fiber‐free enteral formula. He was compliant with all therapies; however, over the next 6 months, he had three admissions with 24 hospital days for altered mental status associated with elevated d‐lactate.

**Figure 1:**
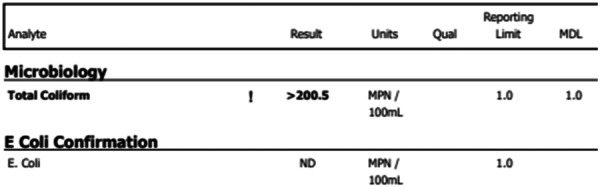
Well water analysis report. mL, milliliter; MPN, most probable number.

The primary team discussed possible fecal transplantation. Concurrently, the family had their well water tested. Testing revealed contamination with coliform, a Gram‐negative bacillus, in amounts >200.5 MPN/100 mL; normal is <1.0 MPN/100 mL (Figure 1). The family switched to drinking bottled water. After removing the contaminated well water from his diet, the patient has not had further episodes of d‐lactic acidosis. He remained on a fiber‐free formula. After 1 year, his central line was removed.

## 2 DISCUSSION

Lactic acid exists in two forms, l‐lactic acid and its enantiomer d‐lactic acid. Humans primarily produce l‐lactate in the anaerobic glycolysis pathway. d‐Lactic acid is endogenously produced in only small quantities via the methylglyoxal pathway.^2^ Both forms of lactate can also be produced by gut bacteria, such as *Lactobacillus* (l‐lactate and d‐lactate), *Streptococcus* (l‐lactate only), and *Bifidobacterium* (l‐lactate only). Patients with SBS are at increased risk for d‐lactic acidosis due to carbohydrate malabsorption and altered gut flora. Unabsorbed carbohydrates are fermented by colonic bacteria leading to an increase in short‐chain fatty acid (SCFA) production. The increase in SCFAs creates a more acidic environment and acts as a substrate for bacterial fermentation. In addition, patients with SBS have altered gut flora including an increase in d‐lactate producing *Lactobacillus*.^3^

d‐lactic acidosis should be on the differential for patients with SBS presenting with undifferentiated AG metabolic acidosis particularly if serum lactate is normal. Diagnosis of d‐lactic acidosis requires separate measurements of d‐lactate levels, ideally drawn during an encephalopathic event. Providers should consider empiric treatment if there is a strong suspicion for d‐lactic acidosis due to variable lab turnaround time.

Acute management of d‐lactic acidosis focuses on correcting the acidosis using acetate‐containing fluids or administering bicarbonate. Lactated Ringers containing l‐lactate and d‐lactate should be avoided. In the acute setting, providers can consider empirically treating thiamine deficiency, which can similarly present with encephalopathy.^4^ Long‐term management focuses on restricting simple carbohydrate exposure, avoiding fermented foods, possible SBBO treatment and as our case highlights, eliminating exposures to environmental contaminants. We also recommend working with a dietician to develop an appropriate dietary plan.^5^

Despite optimizing standard therapy, this patient and family experienced a significant burden related to repeated hospital stays before identifying the contaminated water source. While generally safe, drinking water can expose individuals to pathogenic microorganisms, heavy metals, and organic chemicals. Coliform bacteria, abundant in the natural environment, are generally harmless, but fecal coliforms (such as *Escherichia coli*) can lead to GI illnesses and other long‐term health complications. For this reason, well water that is positive for total coliforms should be avoided. Unlike public drinking water regulated by the US Environmental Protection Agency, private well owners are responsible for their own testing. The Centers for Disease Control and Prevention recommends that private well owners test their water once a year. This case highlights the impact that environmental pollutants can have on human health. Providers should ask about sources of drinking water when managing a patient with symptoms concerning d‐lactic acidosis.

## ACKNOWLEDGMENTS

No funding was received for this manuscript.

## CONFLICT OF INTEREST STATEMENT

The authors declare no conflict of interest.

## ETHICS STATEMENT

Written consent was obtained from the patient's parents for publication of this case report.
